# Subfoveal Choroidal Thickness and Treatment Outcomes of Intravitreal Aflibercept for Branch Retinal Vein Occlusion

**DOI:** 10.3390/life11060572

**Published:** 2021-06-17

**Authors:** Yoshihito Sakanishi, Syu Morita, Keitaro Mashimo, Kazunori Tamaki, Nobuyuki Ebihara

**Affiliations:** Department of Ophthalmology, Juntendo University Urayasu Hospital, 2-1-1 Tomioka, Urayasu City, Chiba 279-0021, Japan; s-morita@juntendo.ac.jp (S.M.); kmashimo@juntendo.ac.jp (K.M.); kztamaki@juntendo.ac.jp (K.T.); ebihara@juntendo.ac.jp (N.E.)

**Keywords:** branch retinal vein occlusion, aflibercept, choroidal thickness, retinal thickness

## Abstract

We aimed to investigate the relationship between subfoveal choroidal thickness (SCT) and treatment outcomes of intravitreal aflibercept (IVA) for macular edema (ME) due to branch retinal vein occlusion (BRVO). We retrospectively evaluated 46 patients with treatment-naive BRVO-ME who underwent IVA treatment between March 2016 and February 2017. There was no significant difference in visual acuity within 6 months (0.29 ± 0.20 vs. 0.27 ± 0.19, *p* = 0.338), the mean central foveal thickness improvement (332.0 ± 162.2 μm vs. 303.9 ± 166.6 μm, *p* = 0.492), and the mean number of IVA injections (1.7 ± 0.7 vs. 1.6 ± 0.7 times, *p* = 0.658) between the SCT thickened (*n* = 26 patients, 26 eyes) and SCT non-thickened groups (*n* = 20 patients, 20 eyes). The rate of ME recurrence was significantly lower in the SCT decreased group (6/17 eyes (35.2%) vs. 19/30 eyes (63.3%); *p* = 0.038). In conclusion, pretreatment choroidal thickening does not affect the therapeutic effect of IVA for BRVO, but ME recurrence was lower in cases of treatment-related choroidal thinning. Thus, changes in SCT may be a therapeutic indicator of IVA for acute BRVO.

## 1. Introduction

Retinal vein occlusion (RVO), the second most common retinal vascular disease after diabetic retinopathy, is a vascular occlusion disease that may cause macular edema (ME), which could ultimately lead to visual loss [1,2,3]. RVO is thought to be associated with hypertension, which affects overall metabolism [4]. RVO is classified according to the vascular occlusion site, either branch retinal vein occlusion (BRVO) or central retinal vein occlusion. Previously, BRVO was primarily treated with retinal photocoagulation based on the results of large-scale studies, but this treatment had limited efficacy [5]. As such, other treatment modalities were introduced, including triamcinolone injection [6] and, more recently, intravitreal injection of anti-vascular endothelial growth factor (VEGF), with the latter currently being the mainstream treatment modality owing to its good results [7,8,9,10,11]. The anti-VEGF drugs approved for BRVO-ME include ranibizumab and aflibercept. Generally, the intravitreal VEGF level in RVO eyes is higher than that in normal eyes [12]. VEGF is believed to be involved in the formation of pathological conditions, which is supported by the effect of anti-VEGF drugs on BRVO-ME. Further, VEGF has a vascular permeability-enhancing effect [13,14]. There are abundant blood vessels not only in the retina, but also the choroid, and studies have shown that choroidal blood flow and thickness influences the outcomes of RVO [15,16,17]. Choroidal thickening in RVO is primarily caused by choroidal vascular hyperpermeability from the increase in intraocular VEGF [18] and by the increase in intraocular nitric oxide [19]. In other words, the change in choroidal thickness may be one of the indicators of intraocular VEGF concentration in eyes with RVO. However, there are no reports on the impact of choroid thickness on the efficacy of intravitreal aflibercept injection (IVA) for BRVO-ME. Thus, this study aimed to investigate the relationship between subfoveal choroidal thickness (SCT) and treatment outcomes of IVA for ME due to BRVO. We discovered that pretreatment of choroidal thickening does not affect the therapeutic effect of IVA for BRVO, but ME recurrence was lower in cases of treatment-related choroidal thinning. 

## 2. Materials and Methods

The subjects were treatment-naive BRVO-ME patients who visited the Juntendo University Urayasu Hospital, Urayasu City, Japan between March 2016 and February 2017, and were treated with IVA for more than 6 months. The eligibility criteria were acute BRVO within 6 months after onset, decimal visual acuity of ≤0.7, and no cataract affecting visual acuity. The exclusion criteria were history of other macular disease, history of vitreous surgery, and history of retinal photocoagulation. Data for the analysis were collected from the patients’ medical records.

The observation period was 6 months, and the injection protocol was IVA once during the induction period and reinjection for ME recurrence or if the central foveal thickness (CFT) remained above 300 μm. Ophthalmic examination was conducted once monthly. The CFT and SCT were measured via optical coherence tomography (OCT) using CIRRUS HD-OCT (Carl Zeiss Meditec, Jena, Germany). CFT was measured at five lines, and SCT was measured at enhanced depth imaging mode. All OCT measurements were performed between 14:00 and 16:00 to lower the effects of diurnal choroidal variation. Best-corrected visual acuity (BCVA) was measured using a decimal acuity chart and converted to logarithm of the minimum angle of resolution (logMAR).

Patients whose SCT of the affected eye was thicker by ≥5% than that of the unaffected eye at baseline were categorized into the SCT thickened group, otherwise they were categorized into the non-thickened group. The number of injections, improvement of BCVA, and CFT for 6 months were examined in each group. In addition, the patients were also divided into two groups according to an SCT decrease of ≥5% in the affected eye at 6 months after the first injection compared with that at baseline (i.e., SCT decreased group and SCT non-decreased group). The amount of BCVA improvement, CFT improvement, and rate of ME recurrence within 6 months were also assessed in these groups.

Between-group comparisons according to SCT thickness were performed using the Mann–Whitney U test. For between-group comparisons, according to SCT decrease, the Mann–Whitney U test was used to assess changes in BCVA and CFT, and the Fisher’s exact test was used to assess the presence of ME recurrence. All statistical analyses were performed using Prism 7 (GraphPad Software Inc., La Jolla, CA, USA), and *p* < 0.05 was considered significant.

## 3. Results

**Figure 1:**
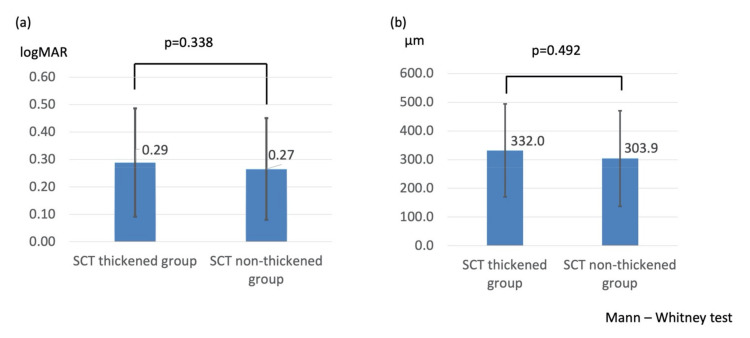
(**a**) Comparison of change in best-corrected visual acuity between the SCT thickened and the SCT non-thickened groups. (**b**) Comparison of change in central foveal thickness between the SCT thickened and the SCT non-thickened groups.

In total, 46 patients were included in the analysis. Of them, 26 patients (26 eyes; mean age, 66.3 ± 9.7 years; 12 males, 14 females) were categorized into the SCT thickened group and 20 (20 eyes; mean age, 66.3 ± 11.4 years; 9 males, 11 females) into the SCT non-thickened group. Table 1 shows the baseline patient characteristics. There were no significant between-group differences in patient characteristics except for SCT. The mean change in BCVA (logMAR 0.29 ± 0.20 vs. logMAR 0.27 ± 0.19, *p* = 0.338, Figure 1a), CFT (332.0 ± 162.2 μm vs. 303.9 ± 166.6 μm, *p* = 0.492, Figure 1b), and number of injections (1.7 ± 0.7 vs. 1.6 ± 0.7 times, *p* = 0.658) within 6 months were also not significant between the SCT thickened and the SCT non-thickened groups.

**Figure 2:**
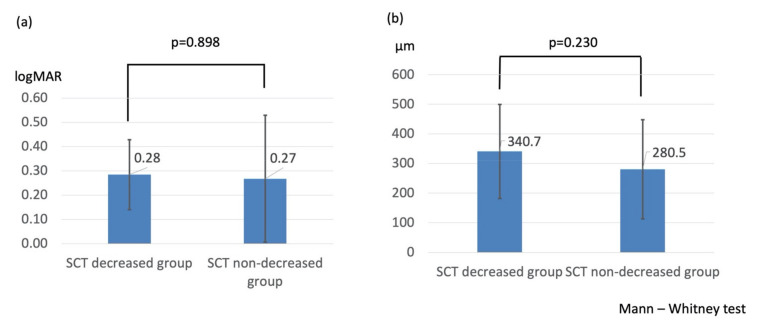
(**a**) Comparison of change in best-corrected visual acuity between the SCT decreased and SCT non-decreased groups. (**b**) Comparison of change in central foveal thickness between the SCT decreased and SCT non-decreased groups.

Similar results were obtained in the comparison between the SCT decreased group and the SCT non-decreased group. There were significant differences in the change of BCVA determined by logMAR (0.28 ± 0.14 vs. 0.27 ± 0.26, *p* = 0.898, Figure 2a), central foveal thickness (340.7 ± 159.2 μm vs. 280.5 ± 167.4 μm, *p* = 0.230, Figure 2b), and incidence rate of ME recurrence (11/30 eyes (36.7%) vs. 11/16 eyes (68.8%), *p* = 0.038) within 6 months between the two groups.

## 4. Discussion

Choroidal thickness at baseline has been reported to be a predictor of the treatment outcomes of IVA for BRVO [16,17]. The results of this study show that pretreatment SCT did not influence the efficacy of IVA, but the risk of ME recurrence was lower in cases where SCT was thinned during the course of treatment.

Hypertension is known to be involved in the onset of RVO, and its relationship to arteriosclerosis has been acknowledged [4]. Hypertension itself is known to affect the choroid [20], but to the best of our knowledge, there are no studies on the effect of treatment for RVO and choroidal thickness, as in our study.

Choroidal thickening in RVO is primarily caused by choroidal vascular hyperpermeability from the increase in intraocular VEGF [18] and nitric oxide [19]. Thus, changes in choroidal thickness may be an indicator of intraocular VEGF concentration in eyes with RVO. There have been several reports regarding the relationship between intravitreal injection of an anti-VEGF drug and choroid thickness and blood flow in BRVO. Okamoto et al. reported that treatment with intravitreal ranibizumab injection (IVR) for BRVO resulted in higher recurrence in patients with thicker foveal choroidal thickness at baseline [16]. In contrast, we found no association between baseline SCT and the number of injections. The difference may be due to the length of the observation period. Okamoto et al. reported the results of a 2-month follow-up, whereas our findings are from a 6-month follow-up.

Hasegawa et al. reported that in IVR treatment in BRVO, the responsive cases had a thicker SCT at baseline in the affected eye than they did in the unaffected eye, whereas there were no significant differences in SCT between the unaffected and affected eyes in the refractory cases [17]. Collectively, these studies indicate that the intraocular VEGF level may be low in patients with treatment resistance and that microaneurysm (MA) had a greater influence than VEGF in refractory cases. A high rate of MA occurring 3 to 6 months after the onset of RVO has been reported [21]. In contrast to the study by Hasegawa et al., all patients in the current study had acute BRVO without MA at baseline and, importantly, SCT at baseline did not influence the efficacy of IVA.

The relationship between intravitreal bevacizumab injection (IVB), an anti-VEGF drug, and choroid thickness remains unclear. One study reported that IVB did not significantly reduce choroidal thickness [22], whereas some studies also reported that choroidal volume is significantly decreased after IVB [23,24]. To our knowledge, there are no reports of choroidal changes in IVA treatment of RVO. However, there are several reports of choroidal changes in IVA treatment of age-related macular degeneration [25,26,27], but the findings have been conflicting. Although some studies found no relationship between the effectiveness of IVA treatment for age-related macular degeneration (AMD) and choroidal change [25,26], Koizumi et al. reported that choroidal changes are associated with the effectiveness of IVA treatment for AMD [27].

IVA has been suggested to reduce choroidal thickness, but it can also be argued that the thinning of the choroid is natural considering the depth of IVA penetration, regardless of the disease. In this study, ME was more likely to be managed with a single injection in patients whose SCT was thinner after IVA treatment than it was before treatment. This means that the effect of IVA includes thinning of the choroid. In contrast, patients who required reinjection because of edema recurrence after injection showed no significant decrease in choroid thickness after treatment. Increased VEGF levels cause choroid thickening through increased nitric oxide production [20]. Thus, ME may recur and choroidal thickness may not diminish in cases in which VEGF levels did not continue to decline after injection. The relationship between ME and choroid thickness can be clarified by investigating the relationship between the timing of ME recurrence and the change in choroidal thickness.

This study has some limitations, including its retrospective design and the small sample size. In addition, this study is concerned with choroidal thickness. However, because choroidal thickness markedly varies among individuals, it cannot be compared between individuals. Moreover, choroidal thickness varies daily [28,29] and decreases with age [30]. However, the same cases were examined to reduce the effect of diurnal variation and age. Comparisons were also made between the affected and unaffected eye per patient. Further large-scale studies of choroidal thickness according to the examination time are needed. In addition, given that the degree of penetration into the choroid differs among the anti-VEGF drugs, further research on IVA is necessary.

## 5. Conclusions

The baseline choroid thickness and change of choroid thickness did not affect treatment outcomes of IVA for acute BRVO. However, the rate of ME recurrence was lower in patients in whom choroidal thinning was related to treatment. Thus, changes in choroidal thickness may be an indicator of the therapeutic effect of IVA for acute BRVO.

## Figures and Tables

**Table 1:** Baseline patient characteristics according to SCT thickness group.

	SCT Thickened Group	SCT Non-Thickened Group	
	n = 26	n = 20	*p*-Value
Age (years)	66.3 ± 9.7	66.3 ± 11.4	0.842
Sex (male/female)	12/14	9/11	1.000
HT (+/−)	16/10	14/6	1.000
Right eye/left eye	15/11	8/12	1.000
BCVA (logMAR)	0.41 ± 0.19	0.31 ± 0.23	0.143
CFT (μm)	564.3 ± 175.2	516.7 ± 135.4	0.236
SCT (μm) Affected eye	266.7 ± 59.1	213.5 ± 54.8	0.004
Unaffected eye	207.7 ± 54.5	236.6 ± 58.2	0.074

Abbreviations: BCVA, best-corrected visual acuity; HT, hypertension; logMAR, logarithm of the minimum angle of resolution; CFT, central foveal thickness; SCT, subfoveal choroidal thickness.

## Data Availability

The data presented in this study are available on request from the corresponding author. The data are not publicly available due to privacy.
